# Effect of Transducer Orientation on Errors in Ultrasound Image-Based Measurements of Human Medial Gastrocnemius Muscle Fascicle Length and Pennation

**DOI:** 10.1371/journal.pone.0157273

**Published:** 2016-06-13

**Authors:** Bart Bolsterlee, Simon C. Gandevia, Robert D. Herbert

**Affiliations:** 1 Neuroscience Research Australia, Sydney, NSW, Australia; 2 University of New South Wales, Sydney, NSW, Australia; Semmelweis University, HUNGARY

## Abstract

Ultrasound imaging is often used to measure muscle fascicle lengths and pennation angles in human muscles *in vivo*. Theoretically the most accurate measurements are made when the transducer is oriented so that the image plane aligns with muscle fascicles and, for measurements of pennation, when the image plane also intersects the aponeuroses perpendicularly. However this orientation is difficult to achieve and usually there is some degree of misalignment. Here, we used simulated ultrasound images based on three-dimensional models of the human medial gastrocnemius, derived from magnetic resonance and diffusion tensor images, to describe the relationship between transducer orientation and measurement errors. With the transducer oriented perpendicular to the surface of the leg, the error in measurement of fascicle lengths was about 0.4 mm per degree of misalignment of the ultrasound image with the muscle fascicles. If the transducer is then tipped by 20°, the error increases to 1.1 mm per degree of misalignment. For a given degree of misalignment of muscle fascicles with the image plane, the smallest absolute error in fascicle length measurements occurs when the transducer is held perpendicular to the surface of the leg. Misalignment of the transducer with the fascicles may cause fascicle length measurements to be underestimated or overestimated. Contrary to widely held beliefs, it is shown that pennation angles are always overestimated if the image is not perpendicular to the aponeurosis, even when the image is perfectly aligned with the fascicles. An analytical explanation is provided for this finding.

## Introduction

The length and pennation of muscle fascicles influence the capacity of skeletal muscles to produce force [1–3]. Consequently biomechanists and muscle physiologists often measure muscle fascicle length and pennation [4–7].

**Fig 1:**
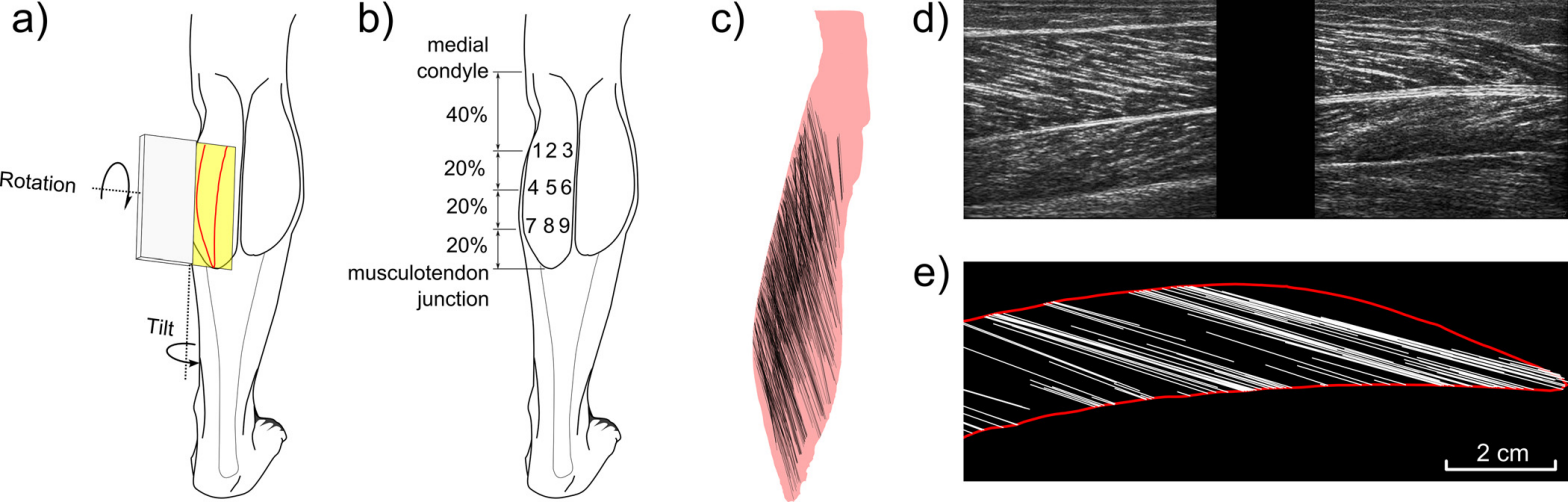
Construction of virtual ultrasound images. (a) Posterior view of the leg. The medial and lateral heads of the gastrocnemius muscle have been outlined. Also shown are the ultrasound transducer (grey box), its corresponding image plane (yellow) and the intersection between the image plane and the medial gastrocnemius surface (red curve). The definitions of transducer ‘tilt’ and ‘rotation’ are indicated by the curved arrows. (b) Sites on the skin at which the virtual transducer was located. (c) Detail of the medial gastrocnemius model (mediolateral view) with fascicles that were reconstructed using DTI data (represented as black lines). (d) Example of a real ultrasound image obtained at the approximate location indicated in (a). The image is orientated so the distal end of the muscle is to the right. The proximal (superficial) and distal (deep) aponeuroses appear as two distinct, nearly horizontal white lines on the image. The fascicles appear as oblique striations with a clear directional pattern. To obtain this image, two ultrasound transducers were rigidly fixed together to form a composite image of 110 × 40 mm with a 18 mm gap (black region) between the images. Note that we do not use real ultrasound images in this study; the image is only used here to show the similarity of real and virtual images. (e) A virtual ultrasound image reconstructed from the same location in the muscle. Virtual images were reconstructed from MRI and DTI data. The aponeuroses (red lines) are calculated as the intersection of the muscle surface with the image plane and the fascicles (white lines) as the part of all 3D fascicles within 2.5 mm of the image plane.

Ultrasonography is widely used to measure muscle fascicle length and pennation in human muscles *in vivo* [8–9]. With conventional ultrasonography the ultrasound transducer is positioned on the skin overlying the muscle, generating a two-dimensional (2D) image of a slice through the muscle. By varying the location and orientation of the transducer, images can be obtained from different parts of the muscle. Aponeuroses and muscle fascicles appear on the images as striations (e.g. Fig 1D). Fascicle lengths and pennation angles can be measured from the images using manual [5] or semi-automated methods [10–11].

To obtain accurate measurements of the length or pennation of a muscle fascicle, the attachments of the fascicle on both aponeuroses must be visible on the image. Accurate measurement of pennation requires, in addition, that the image is perpendicular to the aponeurosis at the point of attachment of the fascicle to the aponeurosis [12]. The first of these criteria (image is aligned with fascicle) is thought to be satisfied when the ultrasound image shows continuous striations extending from one aponeurosis to the other. In practice, it is rarely possible to find a transducer orientation that clearly satisfies this criterion, so the ultrasonographer must manipulate the orientation of the transducer to approximate that criterion as closely as possible. The extent to which the second criterion (image is perpendicular to the aponeurosis) is satisfied is even more difficult to determine. We have shown that when an experienced ultrasonographer obtained ultrasound images from the human medial gastrocnemius muscle under static and passive conditions, the images were misaligned with fascicles by, on average, 5.5° [13]. The same images deviated, on average, 12.1° and 10.6° from the plane perpendicular to the deep and the superficial aponeurosis, respectively (calculated using data presented in [13]). The misalignment is likely to be greater for ultrasound images obtained in dynamic and active conditions.

Some studies have investigated the relationship between the degree of misalignment of an ultrasound image and the size of the error in measurements of muscle fascicle length and pennation [13–16]. Contradictory results have been reported for the gastrocnemius muscle [13, 16]. Bénard et al. [16] compared ultrasound measurements to direct measurements of pennation angles and fascicle lengths of four cadaveric gastrocnemius muscles. They found that moving the transducer away from the plane in which fascicles are oriented caused overestimation of fascicle lengths which, depending on the shape of the aponeuroses, increased either linearly or quadratically with transducer orientation. They did not report a systematic error of pennation angle measurements. In contrast, by comparing to 3D measurements of fascicle lengths and pennation angles from diffusion tensor images, we found that fascicle length measurements from ultrasound images are unbiased but imprecise [13]. This suggests the effect of misalignment of the ultrasound image with muscle fascicles is not straightforward and depends on the 3D geometry of the muscle. The effect of the other requirement for accurate measurements of pennation–having the image plane perpendicular to the aponeurosis–has to our knowledge not yet been investigated at all.

We previously used 3D models of the medial gastrocnemius generated with MRI and DTI to identify transducer orientations which align the ultrasound image plane with fascicles [12]. We found that, while holding the transducer at a single site on the skin, the images could be aligned well with muscle fascicles located in many parts of the muscle simply by applying different combinations of tilt and rotation to the transducer. In that study, we did not quantify the measurement error of fascicle lengths and pennation angles associated with sub-optimal alignment. In the study reported here, 3D muscle models are used to quantify the difference between the actual 3D architectural measures and the 2D values that would be estimated from an ultrasound image. The error is calculated both as a function of the location and orientation of the transducer and as a function of the degree of misalignment with muscle fascicles.

## Methods

This study was conducted by analysing an existing dataset [12–13]. The dataset consists of anatomical MRI and DTI images of the left lower leg of 8 healthy subjects with the ankle in slight dorsiflexion (7°±2°, where 0° means the sole of the foot was perpendicular to the anterior surface of the tibia; S2–S5 Datas). The procedures conformed to the Declaration of Helsinki and were approved by the UNSW Human Research Ethics Committee. Written informed consent of all subjects was obtained prior to their participation. From the MRI data collected on these subjects, 3D geometrical models of the lower leg and the medial gastrocnemius muscle were created (Fig 1). The muscle model includes lines that connect the two corresponding endpoints of muscle fascicles; we refer to these lines as fascicles [12]. On average, 733 fascicles were reconstructed per muscle. The length and pennation was calculated for each fascicle in 3D. The methods used to generate the surface models and muscle fascicles, as well as the methods used to measure fascicle lengths and pennation, have been described in detail elsewhere [12–13].

For the current study, virtual ultrasound images were created from the 3D muscle models. The location and orientation of a virtual transducer was varied systematically to generate a large number of virtual images from different locations in the muscle. On each virtual image, the length and pennation of a single fascicle were measured and compared with the known length and pennation of the same fascicle in the 3D muscle model.

### Virtual ultrasound images

**Fig 2:**
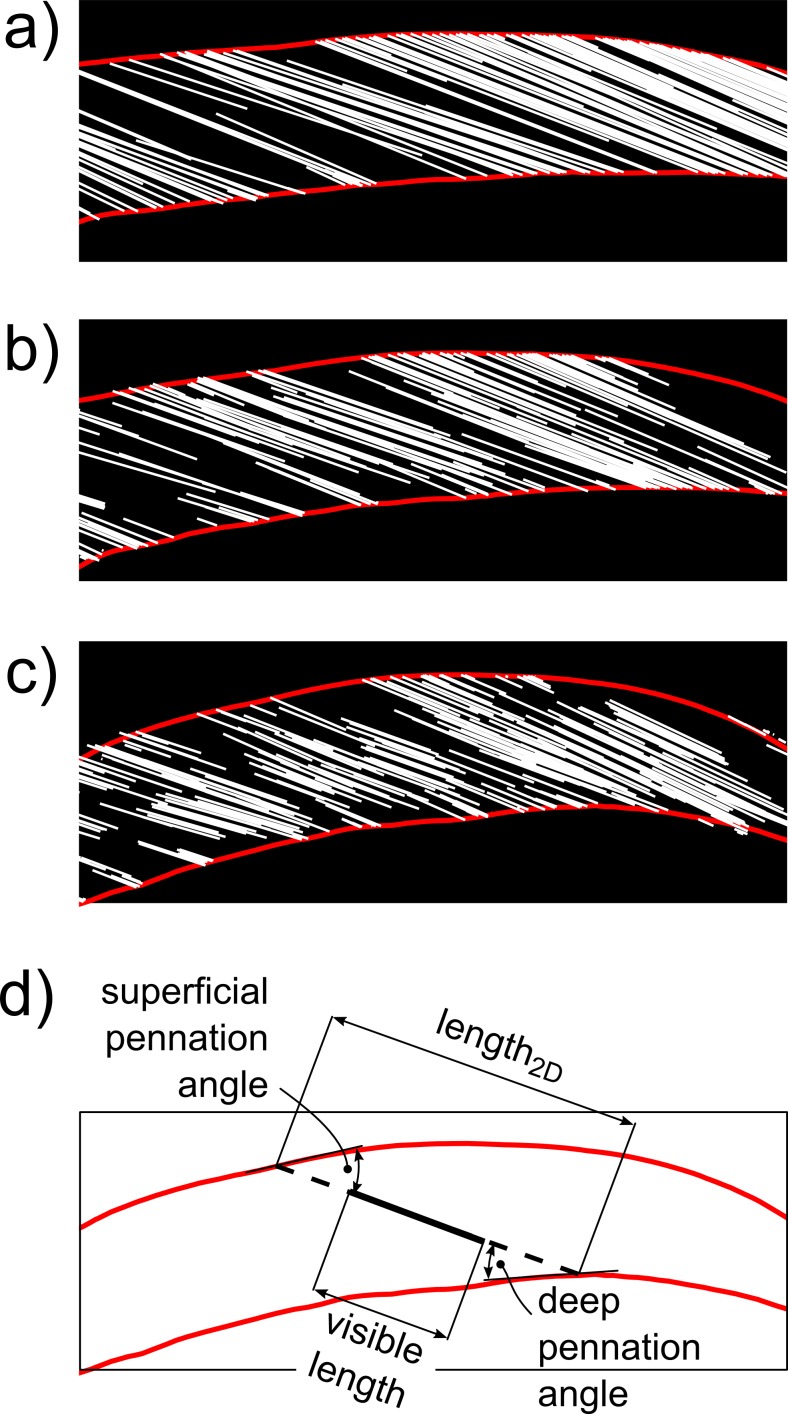
Examples of virtual ultrasound images. Virtual ultrasound images of the medial gastrocnemius obtained with the transducer held at the same site on the skin, but under three different orientations which result in (a) good alignment between image plane and fascicles (misalignment is 1.3°), (b) reasonable alignment (7.5°) and (c) poor alignment (18.3°). Note that the fascicle sections become shorter with increasing misalignment. (d) Same virtual ultrasound image as shown in (c) but with only one fascicle section (solid black line) and its 2D reconstruction (dashed black line), obtained by extending the fascicle section to its intersections with the aponeuroses. For each fascicle section, the length and pennation angle in the 2D image plane were calculated as shown.

The 3D muscle models were used to generate *virtual ultrasound images*. Ultrasound images have a finite thickness of about 5 mm [17]. Consequently the virtual images were comprised of lines representing the parts of aponeuroses and fascicles that were within 2.5 mm of the image plane (Fig 1). The angle between a fascicle and the image plane determines which part of that fascicle is visible: when the angle is 0°, the fascicle is parallel to the image and therefore visible along its entire length, but when the angle is greater the visible section becomes shorter (Fig 2A–2C).

Virtual ultrasound images were created at 9 locations (Fig 1B) and at a range of transducer orientations, as in our previous study [12]. The transducer orientations were defined with respect to a reference orientation in which the transducer is perpendicular to the skin and parallel to the tibia. Virtual images were generated for combinations of tilt and rotation between -40° to +40° in steps of 1° relative to the reference orientation. In the convention used here, “tilt” means rotation of the transducer away from the plane perpendicular to the skin (i.e. decreasing the angle between the image plane and the local tangent plane to the skin) and “rotation” means rotating the transducer away from the parallel to the tibia (Fig 1A; see also [12]). The 9 sites on which the midpoint of the virtual transducer was placed were located on a 3 × 3 grid overlying the belly of the medial gastrocnemius muscle. For the virtual image we chose the same image dimensions of 110 × 40 mm as for real ultrasound images obtained in studies by our group [11, 18]. In total, 472,392 virtual images were created (8 subjects × 9 sites × 81 tilt angles × 81 rotation angles).

### Muscle architecture measurements in 2D

We defined the length of a fascicle as the distance between its attachments on the deep and superficial aponeuroses. In virtual images, both attachments of a single fascicle were only visible in the image when the fascicle was almost parallel to the image plane. Assuming an image thickness of 5 mm and a fascicle of 50 mm with its midpoint exactly in the image plane, the angle between the fascicle and the image plane has to be less than sin^-1^(5/50) ≈ 6° for both endpoints to be visible. To identify the attachments for fascicles which were not perfectly aligned (and which therefore were only partly visible on the virtual image), we applied the same method that is implicitly used when reconstructing fascicles in real ultrasound images [11, 16, 18, 19]: the visible section of a fascicle was extended linearly in both directions until it intersected the superficial and deep aponeurosis (Fig 2). The apparent length of the fascicle, measured from the virtual image, was the distance between the two points of intersection. The pennation angle was calculated as the mean of the deep and superficial pennation angles, defined as the angle in the virtual image plane between the fascicle and vectors tangent to the deep and superficial aponeurosis at their respective intersections with the fascicle.

### Measurement errors

The error in the measurement of length or pennation of a *single fascicle* is the difference between the actual fascicle length or pennation determined from the 3D reconstruction and the 2D measurement obtained from the same fascicle on the virtual ultrasound image. To estimate the error from an *image* in which many fascicles are visible, we calculated the mean absolute error of all fascicles that were visible on the image, weighted by the length of the visible section of the fascicle:

$$mean\;fascicle\;length\;error=\frac{\sum_{i=1}^{i=n}v_{i}∙|{length}_{3D,i}-{length}_{2D,i}|}{n\;\sum_{i=1}^{i=n}\;{length}_{visible,i}}$$ (1)

where *length*_*visible*,*i*_ is the length of the visible section of fascicle *i*; *length*_3*D*,*i*_ and *length*_2*D*,*i*_ are the true 3D fascicle length and the virtual measurement of fascicle length obtained from the virtual 2D ultrasound image, respectively; and *n* is the number of fascicles visible in the image (Fig 1D). The means of both the absolute errors, |*length*_3*D*,*i*_ − *length*_2*D*,*i*_|, and the signed error, *length*_3*D*,*i*_ − *length*_2*D*,*i*_, were calculated. Using the visible length as a weight accounts for the fact that in real ultrasound images the fascicles with the longest visible sections (presenting as long, continuous striations) are those which are most relied upon to determine the course of the muscle fascicles; an error in a longer section is therefore more important than an error in a shorter section. To evaluate the sensitivity of the error to the weighting, we also calculated the non-weighted versions of all error metrics. The errors in pennation angles were defined in a similar way to errors in fascicle lengths.

The purpose of the primary analysis was to determine how muscle architectural errors varied with the degree of misalignment between the ultrasound image plane and the muscle fascicles. The degree of misalignment for any given image was defined as the weighted average of the absolute angles between the image plane and all of the 3D fascicles visible in the virtual image. Again, the lengths of the visible sections of the fascicles were used as weights.

### Effect of misalignment on measurement error

To evaluate the effect of misalignment on errors in measurements of muscle fascicle length and pennation, LOWESS curves were used to regress measurement errors against misalignment for all images from all subjects and sites. (LOWESS is a non-parametric smoother. We applied a first-degree polynomial to 80% of the total data points to calculate the smoothed value for a given point.) This analysis included only images with misalignment less than 10.8° because we had previously found that 95% of (real) ultrasound images are misaligned with the fascicles by less than 10.8° [13]. To investigate the effect of tilt angle on the misalignment-error relationship, separate curves were calculated for tilt angles ranging from -20° to +20°

Data for all 8 subjects, 9 sites and all error metrics (misalignment of image plane with fascicles, and errors in measurement of fascicle lengths and pennation angles), as well as all virtual ultrasound images can be inspected using the Virtual Ultrasound Simulator, a MATLAB-based program to visualise misalignment and error maps for all subjects (S1 Data).

## Results

**Fig 3:**
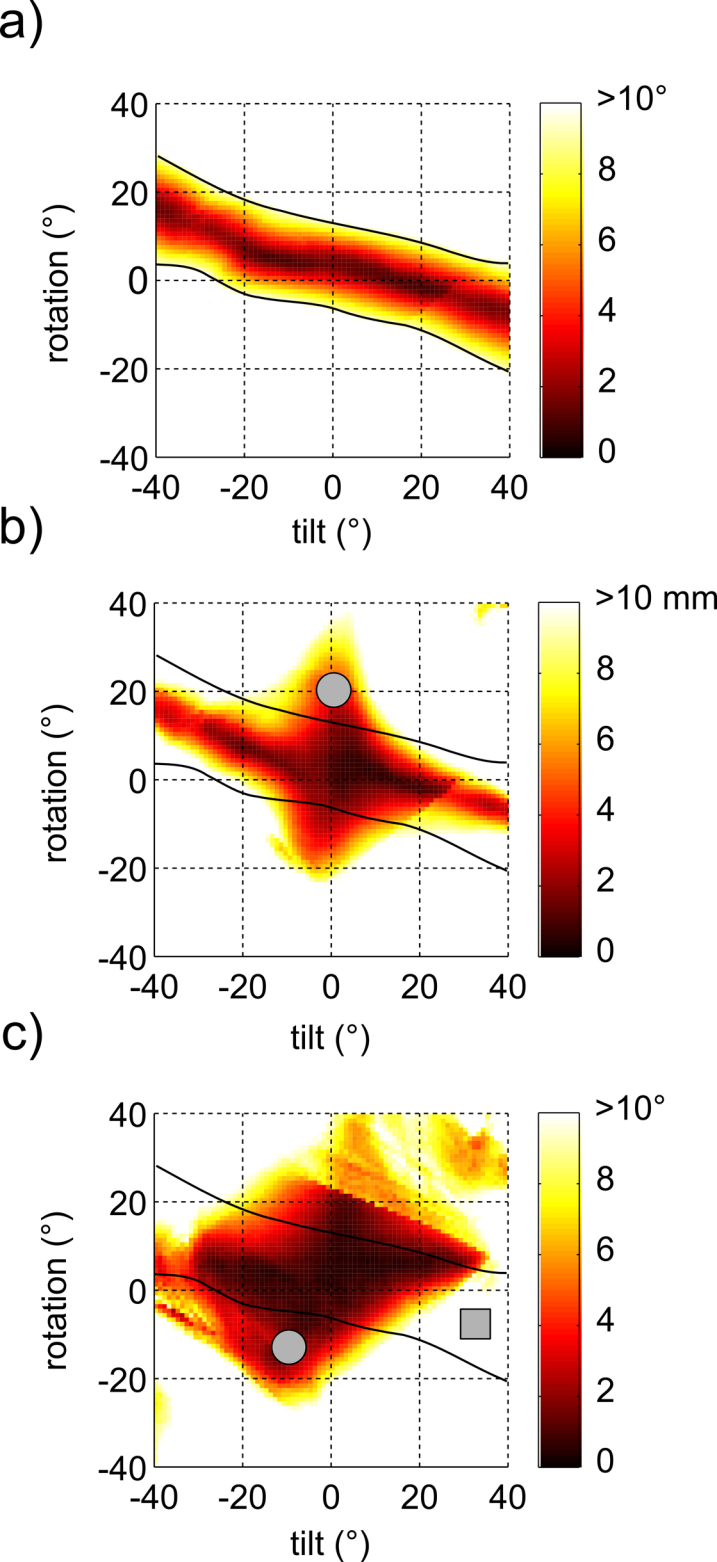
Maps of misalignment and errors in architectural measures. Example of maps of (a) misalignment between the image plane and the fascicles, (b) error in measurements of fascicle length, and (c) error in measurements of pennation angle, as a function of transducer tilt and rotation. All three maps are from one subject and site. The black lines correspond to a misalignment of 10° and are superimposed on all three error maps to show that transducer orientations that result in good alignment (area between the black lines) usually also result in small errors, whereas transducer orientations that result in significant misalignment usually result in large errors. There are some exceptions, as indicated by the grey circles (poor alignment but small error) in (b) and (c), and the square (good alignment but large error in (c). Maps showing similar patterns were found for all subjects at all sites. Data from all subjects can be inspected with the Virtual Ultrasound Simulator (S1 Data).

The reconstruction errors are presented in error maps (Fig 3 and Supporting Information). Each map presents, for one site in one subject, the measurement errors for all evaluated transducer orientations. Thus, the value of a point in the map is the measurement error of an image that was obtained with the virtual transducer at a particular tilt and rotation angle.

**Fig 4:**
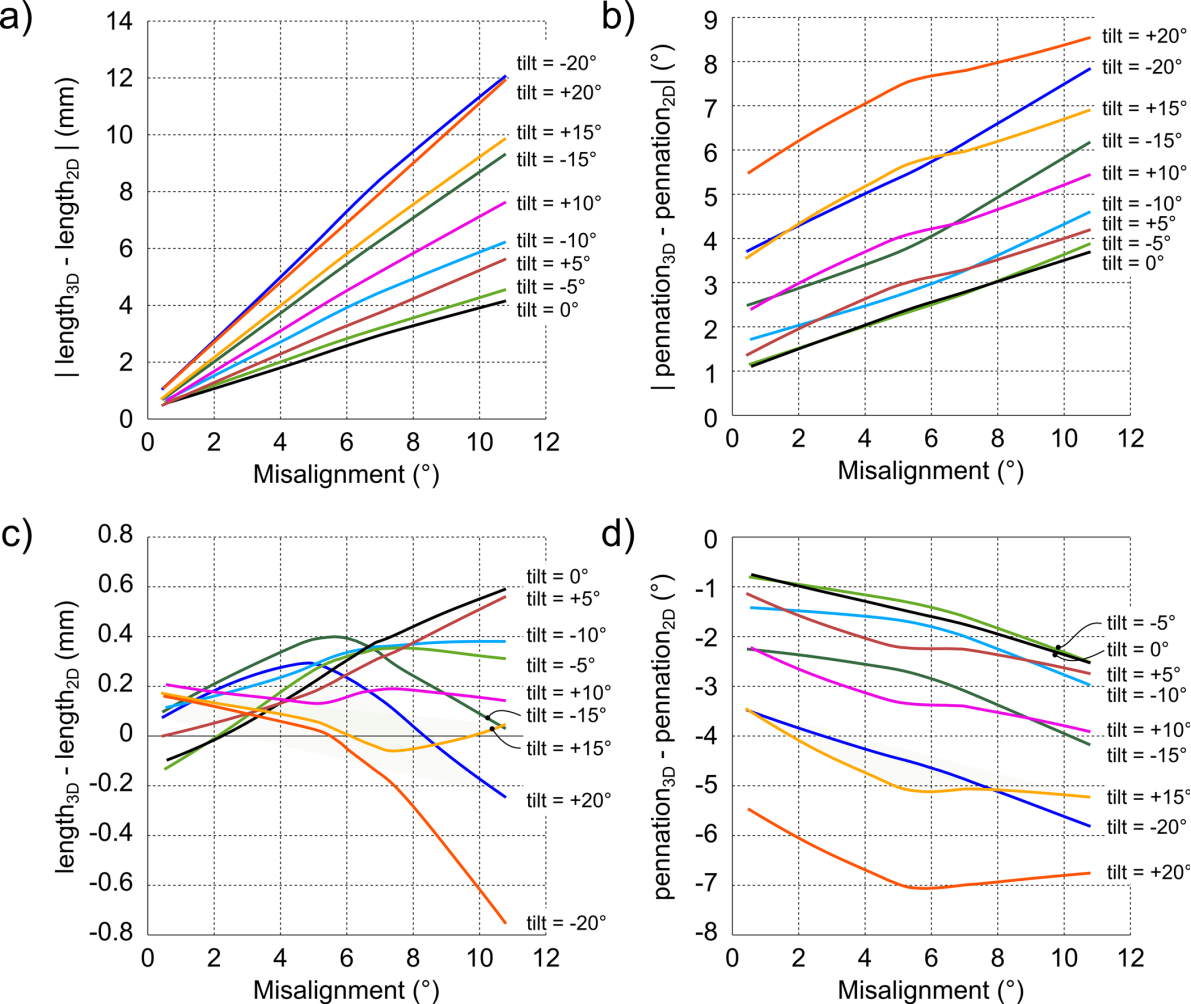
Image misalignment vs. measurement error. Effect of misalignment of the image plane with muscle fascicles on (a) absolute fascicle length error, (b) absolute pennation angle error, (c) signed fascicle length error and (d) signed pennation angle error. The lines are the LOWESS-curves fitted on all misalignment angles lower than 10.8° and their corresponding error values for all subjects and sites combined. Each line presents the LOWESS-curve for one tilt angle between -20° and +20°. Note that an increase in misalignment results in an increase in fascicle length error for all tilt angles (all lines have positive slopes); the smallest increase in error (smallest slopes) occurs at 0° tilt, i.e. when the transducer is held perpendicular to the skin; and mean absolute measurement errors of fascicle lengths approach zero when the transducer is held perpendicular to the skin but mean absolute measurement errors of pennation angle do not.

Taking the data for all sites and subjects together, the relationship between misalignment of the image plane with the fascicle and *absolute* measurement error was linear for both fascicle length and pennation angle: the larger the misalignment, the larger the measurement error (Fig 4A and 4B). However, this relationship depended on tilt angle. Moreover, the dependency of measurement error on tilt angle differed for measurements of fascicle length and pennation angle. For measurements of fascicle length, if the alignment of the image plane with the fascicles was good (misalignment < 1°), the mean absolute error was small (<1.5 mm) for all tilt angles (i.e., the LOWESS-curves converge to near-zero errors at the lowest misalignment). But the slopes of the curves increased from 0.4 mm per degree misalignment at 0° tilt to 1.1 mm per degree at 20° tilt. So, for images of equal misalignment with the muscle fascicles, the image obtained with the transducer perpendicular to the skin resulted, on average, in a smaller fascicle length error than when the transducer was tilted. For pennation, the curves for different tilt angles had similar slopes but different intercepts. Thus, even for images that were aligned well with the muscle fascicles, there was considerable error in measurements of pennation if the transducer was not perpendicular to the skin. At 20° tilt, the error could exceed 5° even if the misalignment of the image plane with the fascicles was less than 1°.

The relationship between misalignment and *signed* fascicle length error did not show a clear pattern and the error was very close to 0 mm (<1 mm) for all tilt angles (Fig 4C). Thus, measurements of muscle fascicle length obtained from virtual ultrasound images did not systematically under- or overestimate true fascicle length. This was not the case for pennation angles. Measurements of pennation obtained from virtual ultrasound images systematically overestimated true pennation (Fig 4D). The extent of the overestimation increased with larger tilt angles.

The error metrics that were weighted by the visible length of a fascicle were very similar to the unweighted metrics: the root mean square difference between the two metrics was 1.1° for misalignment, 1.4 mm for absolute fascicle length error and 1.4° for absolute pennation error.

## Discussion

As expected, ultrasound image-based measurements of muscle fascicle length and pennation of the medial gastrocnemius have least error when the ultrasound image is aligned with the muscle fascicles. For any site on the skin, good alignment between the image plane and fascicles can be achieved by a range of combinations of tilt and rotation (see the dark red region in Fig 4A). Nonetheless the data suggest that the most accurate measurements of muscle fascicle length and pennation will be obtained by keeping the transducer nearly perpendicular to the skin. This conclusion is supported by two observations. First, if the transducer was not perpendicular to the skin, pennation angles were overestimated on ultrasound images even when the image plane was aligned with the fascicles. In practice, this means that pennation angles can be overestimated from perfectly clear ultrasound images on which the full course of fascicles are visible. Second, the effect of misalignment between the image plane and the fascicles on errors in measurements of fascicle length was smallest for images obtained at a tilt angle of 0°. That is, misalignment generated larger errors in measurements of fascicle length when the transducer was not perpendicular to the skin. With a misalignment of 5.5°, the average misalignment of real ultrasound images [13], a typical fascicle length error is just over 2 mm with the transducer at 0° tilt. That error increases to around 4 mm at 10° tilt and almost 7 mm at 20° tilt (Fig 4A).

Based on these considerations, we recommend ultrasound images of the medial gastrocnemius be obtained by orienting the transducer perpendicular to the skin. For measurement of pennation it is more important, at least theoretically, to hold the transducer perpendicular to the *aponeuroses* (instead of the skin). In the medial gastrocnemius, the similarity in curvature between the skin and the aponeuroses means that aligning the transducer perpendicular to the skin makes the image plane nearly perpendicular to the aponeurosis. For other muscles, especially deep muscles such as the soleus, it might be more difficult to find the transducer orientation that intersects the aponeurosis perpendicularly. Our data suggest that in those cases the pennation angle will be overestimated, even if the image is well aligned with fascicles. In the following paragraph we briefly explain this important finding. A more detailed explanation is provided in S1 Text.

**Fig 5:**
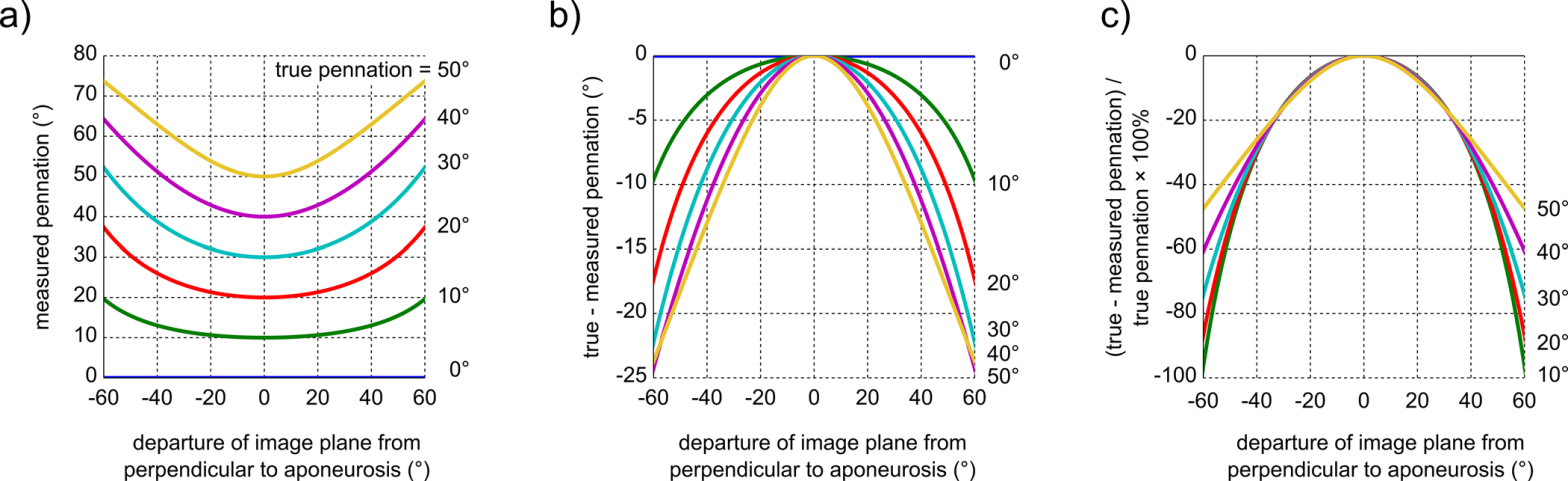
Theoretical error in pennation angle measurements. (a) Measured pennation angle, (b) measurement error and (c) relative measurement error as a function of departure of the image plane from perpendicular to the aponeurosis. The analytical expression used to calculate the curves is provided in S1 Text. Note that pennation angles are always overestimated on ultrasound images, unless the image was obtained perpendicular to the aponeurosis, in which case the pennation is measured correctly. The overestimation does not originate from misalignment between the image plane and fascicles, but solely from not having the image plane perpendicular to the aponeurosis. The curves for 6 values of the true pennation angle ranging from 0° to 50° are shown.

The true pennation angle of a muscle fascicle is the angle between the fascicle and the *plane* that is tangent to the aponeurosis. However, the tangent plane cannot be identified on two-dimensional ultrasound images. Consequently, when ultrasound imaging is used to measure pennation, pennation is defined as the angle between the fascicle and the *line* that is tangent to the aponeurosis. In S1 Text we provide an analytical expression for the error in pennation that arises if the transducer is perfectly aligned with the fascicle but not perpendicular to the aponeurosis. The expression proves that non-perpendicular alignment always leads to overestimation of the true pennation angle. The overestimations are small when the image plane is within 10° of perpendicular to the aponeurosis (error <2%), but increase quadratically with increasing departure from the perpendicular so that large deviations from the perpendicular lead to substantial overestimations of pennation (Fig 5). This has important implications for pennation angles measurements from ultrasound images, because the angle between an aponeurosis and the image plane (and thus the size of the overestimation) is usually not known.

The simulation approach which we have used has advantages and disadvantages. One advantage is that we could evaluate many more images, and under more controlled conditions, than other studies that compare measurements obtained from real ultrasound images with direct measurements made on cadaveric muscle [16, 19, 20]. We calculated reconstruction errors for 472,392 virtual images and on each image we reconstructed many fascicles. This provided a comprehensive description of the relationship between misalignment and reconstruction error and of the effect of transducer alignment on this relationship. Another advantage of the simulation approach is that it minimised the effect of errors in the reference to which the measurements were compared. In our study, the reference was muscle geometry derived from DTI tractography. DTI tractography of muscle is thought to be prone to errors originating from the low signal-to-noise of DTI scans in muscle [21, 22]. However, any errors in the DTI-based muscle geometry would have been replicated in the virtual images; the *difference* between the DTI-based reference and the measurements obtained from virtual images should still be representative of the measurement errors in real images. Note however that other types of errors associated with real ultrasound images are not taken into account here, such as the underestimation of the superficial pennation angle caused by deforming the muscle when pressing the transducer against the skin [13].

**Fig 6:**
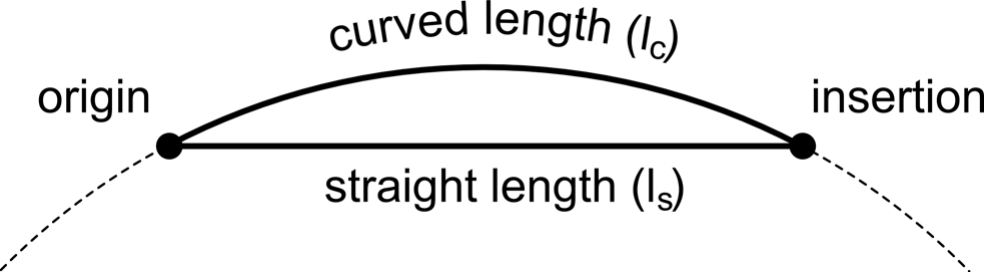
Straight vs. curved fascicle length. Schematic representation of a straight and curved fascicle with the same origin and insertion. The curvature of the fascicle in the figure is exaggerated for presentation purposes. Assuming a constant curvature of 4 m^-1^ along the fascicle length (the fascicle thus lies on the circle with radius 1/curvature indicated by the dashed line), it follows that a fascicle with a curved length of 50 mm has a straight length of 49.9 mm. The straight fascicle is thus only 0.1 mm or 0.2% shorter than the curved fascicle. The curvature of the circle on which the curved fascicle lies is κ.

A disadvantage of our approach is that we used a simplified model of the medial gastrocnemius. The main simplification was to represent fascicles as straight lines between their attachments on the deep and superficial aponeurosis, whereas real fascicles are curved [15, 23, 24]. Muramatsu et al. [24] reported that ignoring fascicle curvature leads to ~6% mean difference in fascicle length measurements. However, they calculated the straight-line-length from the muscle thickness and deep pennation angle and not as the Euclidean distance between the two points of attachment to the aponeuroses, as was done in the present study. The error in fascicle length that originates from disregarding fascicle curvature can be approximated analytically (Fig 6). Consider a fascicle with a length *l*_*c*_ along the fascicle (that is, *l*_*c*_ is the curved length of the fascicle). The straight-line distance between the fascicle’s origin and insertion is *l*_*s*_. Assuming a constant curvature *κ* along the muscle (so that the curved fascicle lies on a circle with radius 1/*κ*), it follows that *l*_*s*_ = 2/*κ*∙*sin*(*κ*∙*l*_*c*_/2). Medial gastrocnemius fascicles are approximately 50 mm long [6] and have curvatures up to 4 m^-1^ at rest [10, 23, 24], so the difference in length is then only 0.1 mm or 0.2%. Even for a very long fascicle of 70 mm and the largest curvature (during a heel raise) of 8 m^-1^ that Darby and colleagues [10] report, the difference is only 0.9 mm or 1.3%. These values are upper limits to the true difference because the difference will be smaller when the curvature is not constant along the course of the fascicle. We therefore expect that our choice to represent fascicles as straight lines, which made our simulations computationally much more efficient, had only a minor influence on the findings.

It is not always straightforward to interpret the information presented on an ultrasound image. We have experienced that the interpretation of images is greatly facilitated by side-by-side comparison of the 3D muscle structure and the part of this structure that is visible on a 2D ultrasound image, as is possible with the Virtual Ultrasound Simulator (Supporting Information). The simulator demonstrates just one way in which detailed muscle reconstructions from MRI and DTI can be useful in the study of muscle architecture. The fusion of data from different sources, each with its advantages and disadvantages, can be used to develop and validate soft tissue models and, as we have done here, to provide guidelines for obtaining measurements of muscle fascicle length and pennation from ultrasound images. Although this study pertained to the human medial gastrocnemius, the findings have implications for ultrasound measurements made on other muscles. The techniques that we and others have developed to measure fascicle lengths and pennation from MRI and DTI are widely applicable and should enable the study of physiological and biomechanical phenomena with greater spatial resolution, albeit lower temporal resolution, than is possible with ultrasound.

## Supporting Information

S1 Data Virtual Ultrasound Simulator (MATLAB-based program). (ZIP)

S2 Data MRI and DTI data for subject 1 and 2. (ZIP)

S3 Data MRI and DTI data for subject 3 and 4. (ZIP)

S4 Data MRI and DTI data for subject 5 and 6. (ZIP)

S5 Data MRI and DTI data for subject 7 and 8. (ZIP)

S1 Text Why pennation angles measured on ultrasound images overestimate true pennation angles. (PDF)
