# Supplementary material for: Effect of Transducer Orientation on Errors in Ultrasound Image-Based Measurements of Human Medial Gastrocnemius Muscle Fascicle Length and Pennation
Source: PLoS One. 2016 Jun 13;11(6):e0157273. doi: 10.1371/journal.pone.0157273 (PMC4905739; doi:10.1371/journal.pone.0157273)
Supplement: S1 Data — (ZIP) [file pone.0157273.s001.zip › user-instructions.pdf]

How to use the Virtual Ultrasound Simulator

Follow these three steps to start the Simulator:

1. Open Matlab and browse to the folder in which the file VirtualUltrasoundSimulator.m is located.
2. Run VirtualUltrasoundSimulator.m by either one of the following options:
    a. Double click on the file to open it and click the ‘Run’-button from the menu bar at the top.
    b. Double click on the file to open it and press F5.
    c. Right-click on the file and select ‘Run’ from the menu
3. Matlab will open a figure window which looks like the figure on the next page.

Notes:

- The Simulator comes with data from 8 subjects, 9 sites per subject and five error metrics. Change to a different subject, site or error metric by using the dropdown-menus. (By default, the Simulator displays the misalignment map for subject 1 and site 5.)
- By default, the reference orientation is displayed (0° tilt and 0° rotation). You can change this orientation by clicking anywhere on the misalignment map in the top-right corner.
- The ultrasound image dimensions can be set to different values than the default of 110 × 40 mm. Note that changing the dimensions will change the virtual ultrasound image and size of the transducers in the 3D figure, but that misalignment and error maps are **<u>not updated</u>**. The misalignment and error maps are only calculated for the default image dimensions. (However, we don’t expect these maps to be much different for other image dimensions.)
- On the virtual ultrasound image, the intersection of the three-dimensional muscle model with the two-dimensional ultrasound image plane in the selected orientation is shown (red line). This intersection can thus be interpreted as the muscle aponeurosis. The (part of) fascicles that are less than 2.5 mm distant to the plane are also displayed (they appear as white lines). That is, it is assumed that all fascicles within 2.5 mm of the image plane are visible on the ultrasound image.
- To rotate the 3D plot, click on the ‘Rotate 3D’-icon (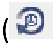) from the menu bar of the figure to switch on rotation mode. Then drag the cursor over the 3D plot to rotate. Note that the rotation-mode should be switched off to select a new orientation from the misalignment map.

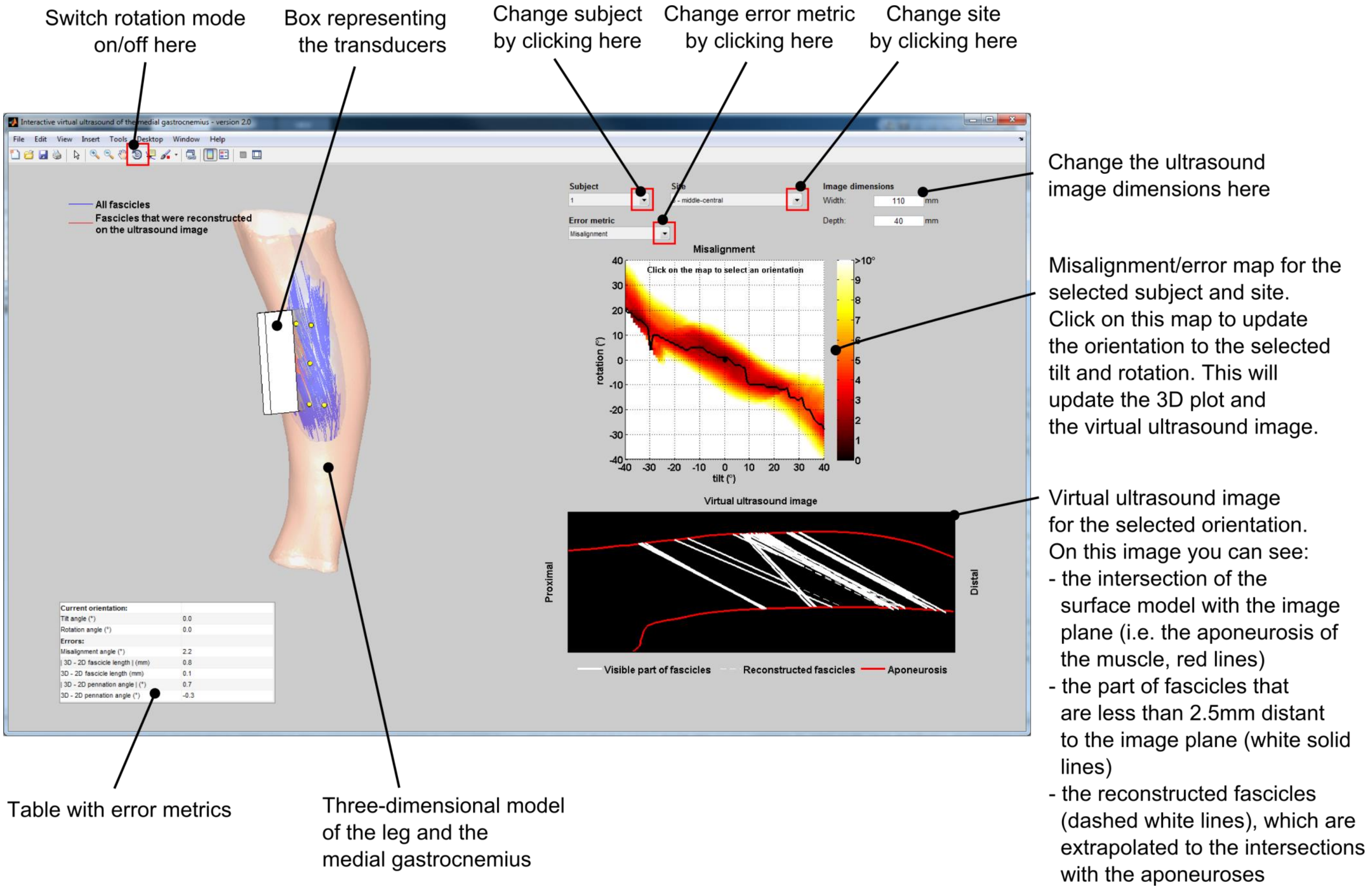

Switch rotation mode on/off here
Box representing the transducers
Change subject by clicking here
Change error metric by clicking here
Change site by clicking here
Change the ultrasound image dimensions here
Misalignment/error map for the selected subject and site. Click on this map to update the orientation to the selected tilt and rotation. This will update the 3D plot and the virtual ultrasound image.
Virtual ultrasound image for the selected orientation. On this image you can see:
- the intersection of the surface model with the image plane (i.e. the aponeurosis of the muscle, red lines)
- the part of fascicles that are less than 2.5mm distant to the image plane (white solid lines)
- the reconstructed fascicles (dashed white lines), which are extrapolated to the intersections with the aponeuroses
Table with error metrics
Three-dimensional model of the leg and the medial gastrocnemius
Interactive virtual ultrasound of the medial gastrocnemius - version 2.0
File Edit View Insert Tools Desktop Window Help
All fascicles
Fascicles that were reconstructed on the ultrasound image
Subject
Site
middle-central
Error metric
Misalignment
Image dimensions
Width: 110 mm
Depth: 40 mm
Misalignment
Click on the map to select an orientation
rotation (°)
tilt (°)
>10°
Virtual ultrasound image
Proximal
Distal
Visible part of fascicles
Reconstructed fascicles
Aponeurosis
Current orientation:
Tilt angle (°) 0.0
Rotation angle (°) 0.0
Errors:
Misalignment angle (°) 2.2
| 3D - 2D fascicle length | (mm) 0.8
3D - 2D fascicle length (mm) 0.1
| 3D - 2D pennation angle | (°) 0.7
3D - 2D pennation angle (°) -0.3
