# Supplementary material for: Effect of Transducer Orientation on Errors in Ultrasound Image-Based Measurements of Human Medial Gastrocnemius Muscle Fascicle Length and Pennation
Source: PLoS One. 2016 Jun 13;11(6):e0157273. doi: 10.1371/journal.pone.0157273 (PMC4905739; doi:10.1371/journal.pone.0157273)
Supplement: S1 Text — (PDF) [file pone.0157273.s006.pdf]

Supplementary material 1

Why pennation angles measured on ultrasound images overestimate true pennation angles

The pennation angle of a fascicle is the angle with which the fascicle attaches to its aponeurosis. Aponeuroses are typically curved three-dimensional surfaces and fascicles are curved lines, so the true pennation angle is the angle between the tangent to the fascicle and the tangent *plane* to the aponeurosis at the fascicle's attachment on the aponeurosis. If an ultrasound image is perfectly aligned with a fascicle, the tangent to the fascicle can accurately be measured from the image. However, a two-dimensional (2D) ultrasound image does not provide the required information to determine the orientation of the tangent *plane*. Instead, the pennation measured from an ultrasound image must be the angle between the fascicle and the tangent *line* to the aponeurosis in the image plane. This tangent line depends on the angle at which the 3D aponeurosis intersects the 2D image plane. The pennation angle that is measured on the image (α_{meas}) will therefore depend on the true pennation angle (α_{true}) and the angle between the image plane and the aponeurosis (θ). Here we derive the function that expresses α_{meas} as a function of α_{true} and θ.

List of symbols

α_{true}	true pennation angle; angle between fascicle and tangent *plane* to the aponeurosis
α_{meas}	pennation angle measured from the ultrasound image; angle between fascicle and tangent *line* to the aponeurosis
θ	angle between the image plane and the perpendicular to the aponeurosis
$\boldsymbol{v_{fasc}}$	fascicle orientation; unit vector parallel to the tangent line of the fascicle at its insertion on the aponeurosis
$\boldsymbol{n_{ap}}$	normal to the tangent plane of the aponeurosis at the insertion of the fascicle
$\boldsymbol{p}$	tangent line to the aponeurosis on the ultrasound image at the insertion of the fascicle; line of intersection between ultrasound image plane and tangent plane to the aponeurosis at the insertion of the fascicle
$\boldsymbol{n_{US}}$	normal to ultrasound image plane

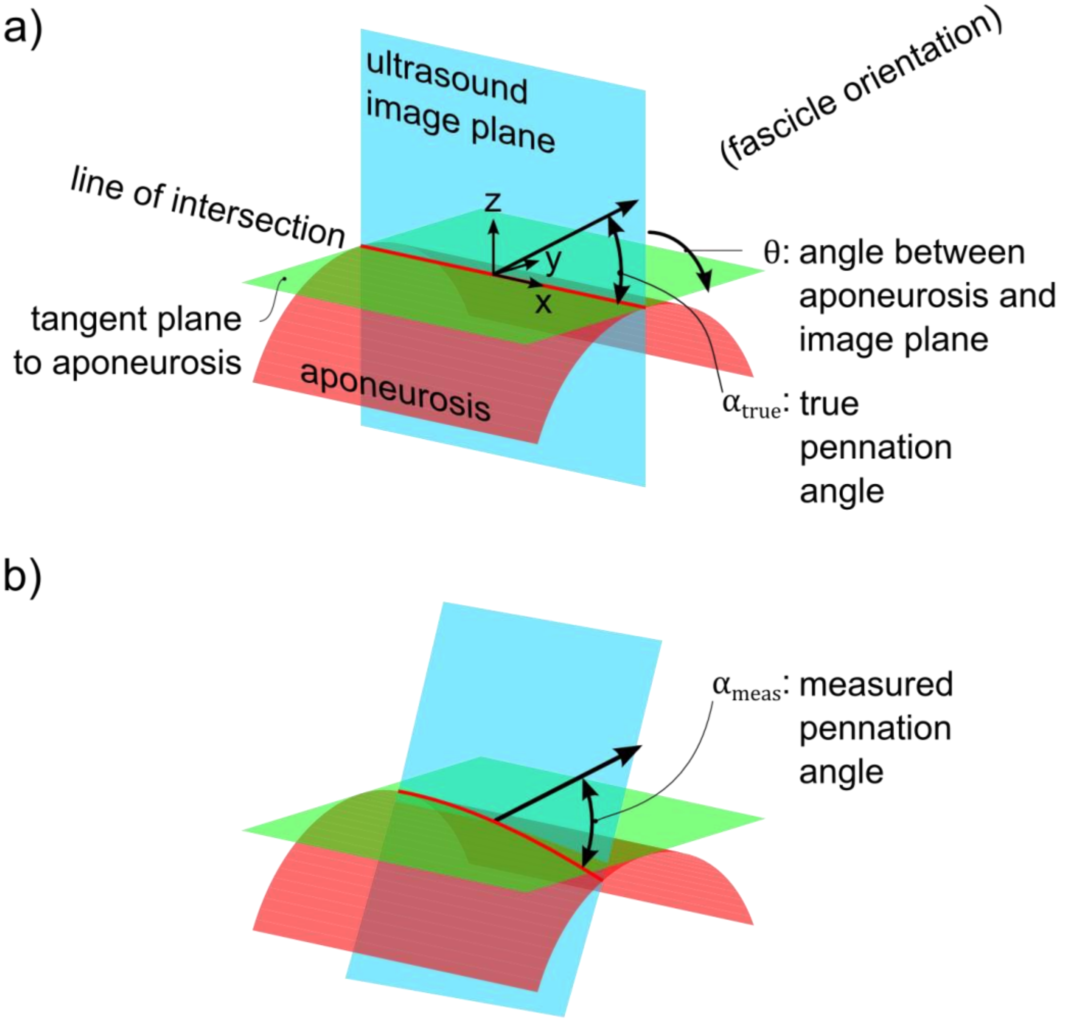

Figure S1 *Schematic representation of an aponeurosis (curved red surface); a tangent line to a fascicle (large black arrow); a tangent plane to the aponeurosis at the point of attachment of the fascicle to the aponeurosis (green plane); and an ultrasound image plane (blue plane). In panel (a), the image plane is perpendicular to the aponeurosis ($\theta = 0°$). Therefore, the angle between the fascicle and the line of intersection of the two planes (the red line, which is the line of the aponeurosis that is visible on the ultrasound image) equals the true pennation angle. Panel (b) shows the same aponeurosis, fascicle and tangent plane, again with the fascicle in the image plane, but in this panel the ultrasound image is not perpendicular to the aponeurosis ($\theta = 25°$). The pennation angle that is measured on the ultrasound image is the angle between the fascicle and the line of intersection between the aponeurosis and the image plane. Although the true pennation angle is the same as in (a), a larger pennation angle will now be measured. Here the aponeurosis is represented as a cylindrical surface, but the same principle applies to aponeuroses with more complex shapes.*

Derivation of the expression

Assume a fascicle that attaches to an aponeurosis under a true pennation angle α_{true}. That is, α_{true} is the angle between the tangent plane to the aponeurosis and the tangent to the fascicle (Fig. S1). Define a coordinate system with the attachment point of the fascicle on the aponeurosis as the origin, the tangent plane to the aponeurosis at the attachment point as the x-y plane and the tangent line to the fascicle at its insertion, $\boldsymbol{v_{fasc}}$, in the x-z plane. The fascicle orientation vector is then $\boldsymbol{v_{fasc}} = [\cos\alpha_{true} \quad 0 \quad \sin\alpha_{true}]^T$. The tangent plane to the aponeurosis has a normal vector $\boldsymbol{n_{ap}} = [0 \quad 0 \quad 1]^T$.

Now assume that an ultrasound image of this fascicle is obtained. To ensure perfect alignment between fascicle and the image, $\boldsymbol{v_{fasc}}$ should be parallel to the image plane. We define a second line that is also parallel to the image plane and that depends on the angle between the ultrasound image and the aponeurosis. This line is located in the y-z plane and makes an angle θ with the z-axis and thus has direction vector $[0 \quad \sin\theta \quad \cos\theta]^{T}$. The angle θ indicates the angle between the image plane and a plane perpendicular to the aponeurosis such that $\theta = 0°$ means that the ultrasound image plane intersects the aponeurosis perpendicularly (Fig. S1a) and any departure from 0° means that the image plane becomes closer to being parallel to the aponeurosis (Fig. S1b). The normal vector to the ultrasound image n_{US} is then:

$$\boldsymbol{n_{US}} = \boldsymbol{v_{fasc}} \times \begin{bmatrix} 0 \\ \sin\theta \\ \cos\theta \end{bmatrix} = \begin{bmatrix} -\sin\alpha_{true} \cdot \sin\theta \\ -\cos\alpha_{true} \cdot \cos\theta \\ \cos\alpha_{true} \cdot \sin\theta \end{bmatrix} \tag{S1}$$

The pennation angle that is measured in the 2D image plane (α_{meas}) is the angle between the local tangent *line* to the aponeurosis and $\boldsymbol{v_{fasc}}$. The tangent *line* lies in the tangent *plane* and is the line of intersection between the ultrasound image plane and the tangent plane. The tangent line to the aponeurosis in the image plane $\boldsymbol{p}$ is the null-space of the matrix composed of the normal vector to the ultrasound plane and the normal vector to the aponeurosis' tangent plane:

$$\boldsymbol{p} = null\left(\begin{bmatrix} \boldsymbol{n_{US}}^{T} \\ \boldsymbol{n_{ap}}^{T} \end{bmatrix}\right) = \begin{bmatrix} -\cos\alpha_{true} \cdot \cos\theta \\ \sin\alpha_{true} \cdot \sin\theta \\ 0 \end{bmatrix} \tag{S2}$$

The measured pennation angle α_{meas} is the acute angle between $\boldsymbol{p}$ and $\boldsymbol{v_{fasc}}$ and therefore is:

$$\alpha_{meas} = \cos^{-1}\left(\frac{\boldsymbol{p}\cdot\boldsymbol{v_{fasc}}}{\|\boldsymbol{p}\|\|\boldsymbol{v_{fasc}}\|}\right) = \cos^{-1}\left(\frac{\cos^2\alpha_{true}\cdot\cos\theta}{\sqrt{\cos^2\alpha_{true}\cdot\cos^2\theta+\sin^2\alpha_{true}\cdot\sin^2\theta}}\right) \quad \text{(S3)}$$

Difference between true and measured pennation angle

Substituting $\theta = 0°$ in Eq. S3 gives the result $\alpha_{\mathrm{meas}} = \alpha_{\mathrm{true}}$, which means that with the image plane perpendicular to the aponeurosis, the true pennation angle is correctly measured from the ultrasound image. It also follows from Eq. S3 that when $\theta \neq 0°$ the measured pennation angle is always larger than the true pennation ($\alpha_{\mathrm{meas}} > \alpha_{\mathrm{true}}$). That is, the ultrasound measured pennation angle is an overestimation of the true pennation if the image plane is aligned with the fascicle but does not intersect the aponeurosis perpendicularly.

Using Eq. S3, α_{meas} was calculated for α_{true} ranging from 10° to 50° (with steps of 10°) and θ ranging from -60° to +60°. The measurement error was then calculated as the difference between the true and the measured pennation angle ($\alpha_{true} - \alpha_{meas}$), and the relative measurement error as the error expressed as percentage of the true pennation angle $(\alpha_{true} - \alpha_{meas})/\alpha_{true} \times 100\%$ (Fig. 5 in the main text). The overestimation increases approximately quadratically with increasing departure from the perpendicular, and approximately linearly with increasing true pennation angle (Fig. 5b). The relative error is more or less constant for different values of true pennation angles (Fig. 5c), especially between -30° and +30° angle of departure from the perpendicular.
